# Cassava starch enhances the printability and swallowability of rapeseed protein gels for 3D-printed dysphagia foods

**DOI:** 10.3389/fnut.2026.1888256

**Published:** 2026-09-15

**Authors:** Yuxing Zhang, Jingru Dong, Yike Su, Xiaoqing Xiao, Siting Li

**Affiliations:** College of Food Science and Technology, Hunan Agricultural University, Changsha, China

**Keywords:** 3D printing, cassava starch, dysphagia, food inks, rapeseed protein

## Abstract

Plant-based proteins have attracted increasing attention in the development of easy-to-swallow foods and three-dimensional printing (3DP) materials due to their excellent nutritional properties. However, the disordered arrangement of protein molecular chains in rapeseed protein (RP) results in insufficient structural stability and poor self-supporting capacity during 3DP, leading to collapse behaviour. To address this limitation, cassava starch (CS) was incorporated into RP gels at different CS: RP ratios to improve printability and swallowing properties. FT-IR and XRD analyses demonstrated that the addition of 1%–5% CS promoted hydrogen bonding interactions with RP, thereby enhancing the gel network structure and increasing the storage modulus. Texture analysis revealed that the obtained gels possessed suitable cohesiveness and hardness (28.67–36.33 g), indicating favorable swallowability characteristics. Moreover, the incorporation of CS significantly improved the viscosity, printing fidelity, and self-supporting strength of RP gels, effectively eliminating collapse during the printing process. Among all formulations, the 2%-CS group exhibited the optimal printing performance, achieving the highest storage modulus, the lowest creep strain (3.06%), and a printing accuracy of 97.37%. In addition, this formulation satisfied the requirements for Level 4 foods according to the International Dysphagia Diet Standardisation Initiative (IDDSI). LF-NMR analysis further indicated that the enhanced properties originated from the water-retaining network formed through cross-linking interactions between RP and CS. Overall, low concentrations of CS effectively improved the printability and swallowability of RP gels, providing a scientific basis for the development of 3D-printed dysphagia-friendly foods.

## 1 Introduction

With the rapid acceleration of global population aging, dysphagia has emerged as a significant public health priority (1). Epidemiological data indicate a prevalence reaching up to 60% in specific demographics, with incidence rates rising sharply with age (2). For the majority of older adults, achieving safe and efficient nutritional intake remains a formidable challenge, typically necessitating the consumption of pureed, minced, or moist foods. However, the frequent blandness and low sensory appeal of traditional texture-modified diets often diminish patient compliance and appetite. Consequently, engineering food systems that harmonize swallowing safety, nutritional density, and aesthetic appeal has become a focal point of food science.

3D printing (3DP), an additive manufacturing technology based on layer-by-layer deposition, offers a transformative approach for the personalised fabrication of complex food structures (3). Currently, 3DP is catalyzing innovations in functional carriers, fat substitutes, and specifically, texture-modified foods for individuals with dysphagia. By enabling precise customisation of geometry and internal architecture, 3DP significantly augments the visual and sensory appeal of paste-like foods, thereby stimulating appetite (4). Simultaneously, the systematic optimisation of 3DP formulations allows for the precise modulation of rheological properties and nutritional composition, ensuring that meals are both nutritionally adequate and safe for swallowing.

Printable gel systems represent the most promising candidates for 3DP inks, requiring a delicate balance of shear-thinning behaviour and self-supporting capacity (5). Given their superior nutritional profile and sustainability, plant proteins have become a primary focus for ink development. Rapeseed protein (RP), a complete protein comprising napin and cruciferin, is recognized as a high-quality dietary source due to its balanced essential amino acid profile, comparable to milk and soy protein, and its high levels of lysine, valine, and proline (6, 7). Cruciferin is composed of multiple polypeptide chains, and non-covalent interactions mainly maintain its structural conformation. In contrast, napin is composed of two polypeptide chains, and its structural stability mainly depends on disulfide bonds. The differences in molecular structure and structural stabilization between the two lead to distinct thermal and functional characteristics. These differences may lead to its interaction with polysaccharides and affect the formation of complex gel networks. However, under neutral pH, RP exhibits poor solubility and limited interfacial adsorption, resulting in weak gelation and inadequate mechanical strength (8). This often leads to post-printing structural collapse, severely restricting its application in 3DP. Compared with plant protein systems such as soybean protein and pea protein, the application research of RP in easy-to-swallow food is still relatively limited, and its functional characteristics also have certain limitations, which provides research space for further exploring the application of RP in easy-to-swallow food. Although methods to modify printability have been developed, such as acid-emulsified fibrillation (9) and protein-polyphenol complexation (10, 11), these techniques are frequently timeconsuming and logistically complicated. The incorporation of polysaccharides into protein systems is a well-established strategy for modulating the physicochemical properties of hybrid gels.

Polysaccharides can induce protein unfolding and cross-linking, thereby altering the network density and water-holding capacity (WHC) of the system. This subsequently improves ink adaptability and forming precision during printing procedures (12). The textural characteristics of proteins are affected differently by different polysaccharides, which determines the application scenarios for 3DP. Researchers have conducted in-depth investigations in domains that require high-strength gel structures: Guo et al., for example, added the dietary fibre polysaccharide inulin to quinoa protein to deliver curcumin. They improved the ink’s compactness by altering hydrophobic interactions, and its denser gel texture ensured intestinal release of curcumin (13). Furthermore, myofibrillar proteins and trehalose have been combined to create 3D-printed HIPES that prevent ice crystal formation and provide mechanical support for freeze–thaw stability (14).

Although polysaccharides can provide good mechanical support for 3DP, the aforementioned highstrength polysaccharide gel systems have limitations for use in easily swallowed foods. Patients with difficulty swallowing have specific requirements for meal texture, and high-strength polysaccharide gels may make chewing more challenging. Cassava starch (CS), a widely available, low-cost natural polysaccharide with a mild taste, exhibits excellent thickening, gelling, and water-holding capabilities. The tensile strength of CS is only 430 kg/m^2^, while that of kudzu root and sago starch is roughly 1,500 kg/m^2^ (15). As a result, CS gels have a softer texture, making them easier to disintegrate in the mouth than other starches. It has a special potential to create foods that are easier to swallow (16). Meanwhile, several studies have shown that adding proteins to soft CS can enhance its extrusion flowability, making it a suitable material for 3DP (17, 18). Therefore, by utilising protein-polysaccharide interactions, adding CS to the RP gel system shows promise for satisfying the dual goals of swallowing safety and 3D printability. The interactions and underlying mechanisms between RP and CS molecules remain unknown, and there is currently a lack of studies on combining RP and CS for 3DP, particularly for easily swallowable foods. Therefore, this study investigated the rheological behaviour, textural properties, moisture distribution, and microstructure of CS-RP hybrid gels under different CS ratios. Furthermore, through 3DP and IDDSI (International Dysphagia Diet Standardisation Initiative) testing, it elucidated the relationships among these properties, printing precision, and swallowing safety. By optimising the composite system, this study aims to develop a novel food ink with high print accuracy and sensory acceptability for individuals with dysphagia. This approach not only opens new avenues for the high-value utilisation of RP but also provides a theoretical and technical foundation for the personalised customisation of nutritional foods.

## 2 Materials and methods

### 2.1 Materials

Cassava starch (CS, molecular weight: 1.98 × 10^7^ g/mol, purity ≥99%) was purchased from Shanghai Yuanye Technology Co., Ltd. Rapeseed protein isolate (RP, 90% protein content on a dry basis) was provided by Shanxi Baohe Biotechnology Co., Ltd. All other chemical reagents used in our experiments were of analytical grade.

### 2.2 Preparation of CS-RP hybrid gels

CS-RP hybrid gels were prepared as follows. The solid content of each sample was fixed at 15% w/w. CS (0, 1, 2, 5, 7, 9, and 15% w/w) was mixed with RP, suspended in purified water, and stirred magnetically for 30 min at 90 °C (YuRui Instrument Manufacturing Co., Ltd., Changzhou, Jiangsu, China). The mixture was then refrigerated at 4 °C for 30 min to form the gel. This process promotes starch water absorption and facilitates the formation of a stable three dimensional gel network with the protein. During the heating process, no additional water replenishment or quality correction is performed to account for water loss due to evaporation; therefore, 15% (w/w) is the nominal total solids content based on the initial formula calculation. The resulting gels were labelled 0%-CS, 1%-CS, 2%-CS, 5%-CS, 7%-CS, 9%-CS, and 15%-CS, respectively.

### 2.3 3DP process

Using the FOODBOT-EMCN Coaxial Extruded Food 3D Printer (Hangzhou Panda Technology Co., Ltd., China) connected to Simplify3D software version 4.0.1, CS-RP hybrid gels were printed at room temperature (25 °C). The 3D printer parameters were set as follows: nozzle diameter of 0.8 mm, extrusion line width of 0.96 mm, printing speed of 23 mm/s, and infill density of 96%. The calculation of printing accuracy references the method proposed by Bao (19):

$$\begin{array}{l} \text{Printing accuracy}(\%)=\\ \;\;\;\;\frac{\left(1-\frac{|L-\mathrm{Lm}|}{\mathrm{Lm}})+(1-\frac{|\mathrm{He}-\mathrm{Hm}1|}{\mathrm{Hm}1})+(1-\frac{|\mathrm{Hc}-\mathrm{Hm}2|}{\mathrm{Hm}2}\right)}{3}\times 100\% \end{array}$$ (1)

Where L, He, and Hc denote the printed sample length, edge height, and centre height (mm), respectively, whereas Lm, Hm1, and Hm2 denote the length, edge height, and centre height (mm) of the 3D model in STL format. Finally, ImageJ software was used to measure the length, line width, edge height, and centre height of the 3D-printed samples, thereby computing the printing accuracy.

### 2.4 Rheological properties

The rheological properties were examined using a Discovery HR10 hybrid rheometer fitted with parallel plates measuring 40 mm in diameter and 1 mm apart. The ambient temperature was maintained at 25 °C during the experiment to systematically assess the CS-RP hybrid gel’s potential as a printing material. All tests were conducted three times.

#### 2.4.1 Shear rate sweep test

A shear rate range of 0.1 to 100 s^−1^ was used to test the apparent viscosity of CS-RP hybrid gels. The collected viscosity data were fitted using the power-law model for additional investigation (20):

$$\eta =K\times \gamma^{n-1}$$ (2)

Here, η, K, γ, and n stand for the printing ink’s viscosity (Pa·s), consistency index (Pa·s^n^), shear rate (s^−1^), and power-law exponent, respectively.

#### 2.4.2 Shear frequency sweep testing

To describe the rheological characteristics of the gel as it leaves the printing nozzle, we swept angular frequency from 1 to 100 rad/s while operating in the linear viscoelastic region (LVR) at a fixed strain of 1.0%. We calculated the storage modulus (*G’*), loss modulus (*G”*), and loss factor (*tan* δ) for every sample.

#### 2.4.3 Creep testing

The creep recovery characteristics were measured using a method adapted from Riantiningtyas (21). First, a constant shear stress of 10 Pa was applied to the sample and maintained for 3 min (creep phase). Subsequently, the stress was rapidly removed, and the strain recovery process was recorded for 7 min (recovery phase). All tests were conducted within the material’s LVR, with each sample undergoing two complete cycles to ensure the reliability of the results. The percent recovery during the stress removal phase is calculated as follows:

$$\text{The percent recovery}(\%)=\frac{\varepsilon 0-\varepsilon 1}{\varepsilon 0\;}\times 100\%$$ (3)

In the recovery phase, the strain values at 180 and 420 s are denoted by ε0 and ε1, respectively.

### 2.5 Texture profile analysis (TPA)

To simulate the oral chewing process and quantify its textural properties, this study conducted two consecutive compression cycles on gel samples at 25 °C using a Brookfield CT3 texturometer (Brookfield Polymer, USA), equipped with a cylindrical probe (Model TA5) and a fixture (TA-RT-KI). The probe compressed the sample twice at a constant speed to 50% of its initial height, as described by Liu et al. (22). With a trigger load of 5 g, the pre-test and test speeds were both 2 mm/s. This test quantifies texture qualities, including cohesiveness, hardness, chewiness, and adhesiveness. All tests were repeated three times.

### 2.6 Low-field nuclear magnetic resonance (LF-NMR)

A nuclear magnetic resonance (NMR) analyser (model NMI20-015 V-I, NuMag Co., Ltd., Suzhou, China) was used to measure the gel relaxation time. To produce each sample, 2 g of gel was weighed, carefully wrapped in thin plastic film, and three replicates were set up for each group. Before being inserted into the NMR device for analysis, the prepared samples were placed in glass tubes with an inner diameter of 50 mm. The Carr–Purcell–Meiboom–Gill (CPMG) pulse sequence was used to capture the spin–spin relaxation time. The following parameters were set: time wait (TW) = 4,000 ms, time echo (TE) = 0.40 ms, number of echoes (NECH) = 8,000, number of scans (NS) = 8. 10^6^ iterations were then used to invert the acquired raw data. To guarantee reproducibility, each test was conducted three times.

### 2.7 Fourier transform infrared spectroscopy (FT-IR)

First, lyophilised specimens were ground into a fine powder. A thin, transparent pellet was formed by uniformly blending 1 mg of the resultant powder with 100 mg of potassium bromide (KBr) for every measurement. A Shimadzu IRTracer-100 spectrometer (provided by Zhengzhou Fanrui Yunzhi Technology Co., Ltd.) with a resolution of 4 cm^−1^ and 32 cumulative scans was used to measure absorbance over 400–4,000 cm^−1^.

### 2.8 X-ray diffraction (XRD)

An XRD (Rigaku MiniFlex 600, Japan) was used to evaluate the crystalline structure of the CS-RP hybrid gel after the freeze-dried samples were ground into powder. The scanning rate was set to 10°/min, and the 2θ range was set to 5 °–60 °.

### 2.9 Scanning electron microscope (SEM)

The sample was freeze-dried and gold-sputtered, and its microstructure was examined using an SEM at 5 kV. Scale bars of 100 μm and 200 μm were used to acquire the images. A Tescan-Mira4 (Tianjin Yanta Technology Co., Ltd., China) was used.

### 2.10 IDDSI

Samples (24 mm × 28.8 mm × 8 mm) were evaluated following the IDDSI framework (23). In the Fork Pressure Test, thumb pressure was applied until the fingernail blanched; surface indentation and sample recovery were recorded. In the Spoon Tilt Test, a sample-filled spoon was tilted at 45° (usage angle metre auxiliary visual measurement control angle) for 10 s to evaluate flowability and residue, and samples were categorised as IDDSI Levels 4 (Pureed) or 5 (Minced & Moist). All IDDSI tests were repeated multiple times to ensure consistency of results, but they were not conducted under blinded conditions.

### 2.11 Statistical analysis

Experimental results were analysed using IBM SPSS (version 26.0) to conduct a one-way analysis of variance (ANOVA) and test for significance (*p* < 0.05). Data visualisation was performed using Origin 2026 and GraphPad Prism (version 10.0) software.

## 3 Results and discussion

### 3.1 Printability of CS-RP ink

Samples were 3D printed in a cubic shape measuring 24 mm × 28.8 mm × 8 mm to assess the printability of CS-RP ink at various concentrations. This study analysed parameters including length, line width, edge height, centre height, and print accuracy (as shown in Figure 1 and Figure 2). The results reveal the performance characteristics of the printing ink, including support stability and shape retention.

**Figure 1:**
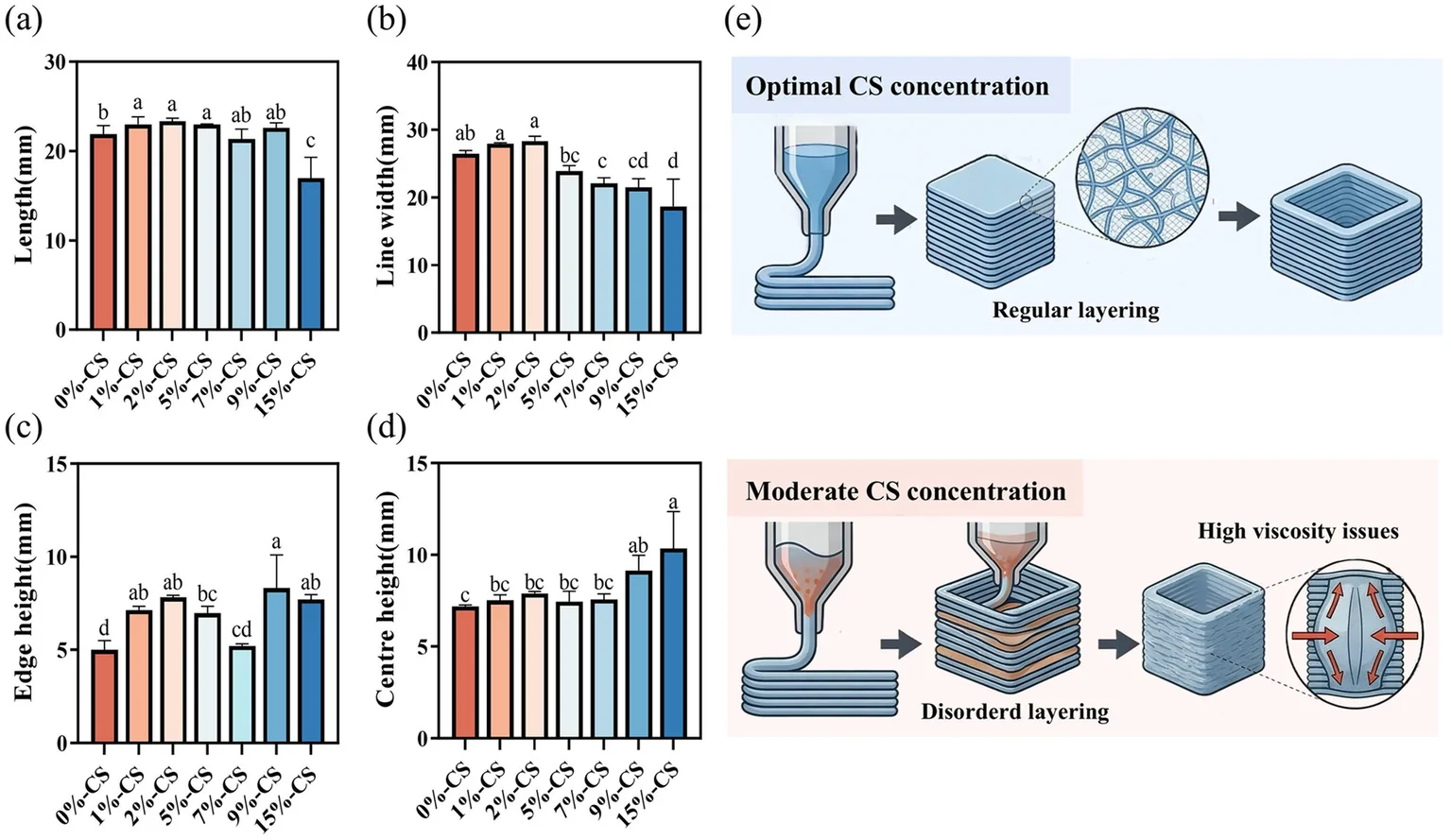
Dimensions of 3D-printed samples with different CS content formulations **(a)** length; **(b)** line width; **(c)** edge height; **(d)** centre height; **(e)** schematic diagram of the printing process. The error bars represent the standard deviation of the mean. Different letters indicate statistically significant differences between samples (*p* < 0.05). Samples are labelled 0%-CS, 1%-CS, 2%-CS, 5%-CS, 7%-CS, 9%-CS, and 15%-CS, representing CS content of 0, 1, 2, 5, 7, 9, and 15% w/w in the CS-RP hybrid gels (total solids content 15% w/w).

**Figure 2:**
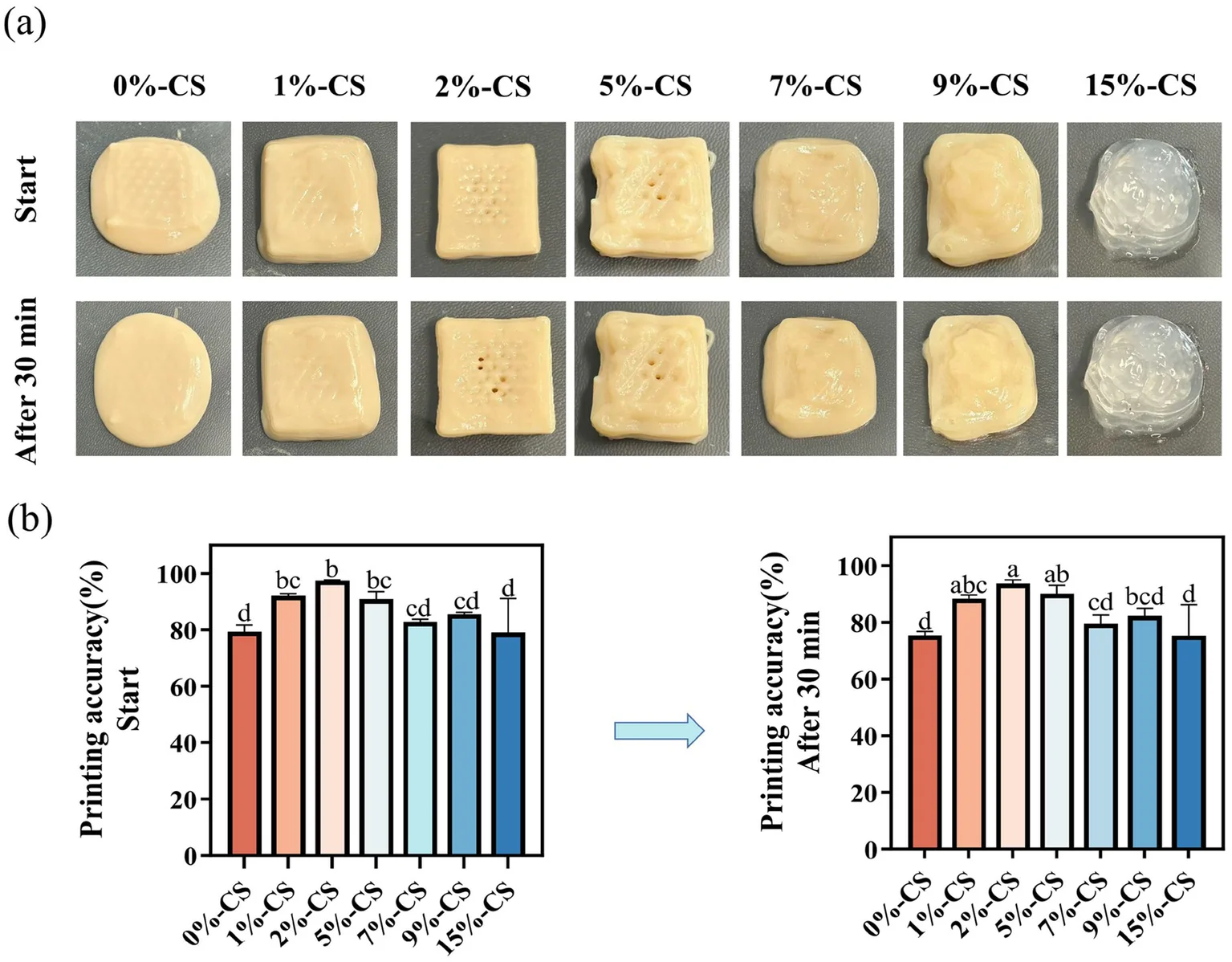
All 3D-printed products **(a)** printability of different CS-RP hybrid gel formulations and changes after 30-min storage **(b)** printing accuracy of different CS-RP hybrid gel formulations at formation and after 30-min storage. The error bars represent the standard deviation of the mean. Different letters indicate statistically significant differences between samples (*p* < 0.05). Samples are labelled 0%-CS, 1%-CS, 2%-CS, 5%-CS, 7%-CS, 9%-CS, and 15%-CS, representing CS content of 0, 1, 2, 5, 7, 9, and 15% w/w in the CS-RP hybrid gels (total solids content 15% w/w).

As shown in Figure 1, the edge height of the control group 0%-CS was 5.00 mm, which was much lower than the model’s 8.00 mm. Concurrently, printing collapsed, exhibiting inadequate selfsufficiency. This resulted from the RP gel network’s inadequate mechanical strength, which makes it difficult for the network to support its own weight (24). The addition of CS significantly reduced this collapse. During printing, every composite gel held its original shape effectively. With a strong overall structure, a smooth surface, and clearly defined edges, the 2%-CS gel most closely resembled the model in terms of print quality. After printing, it displayed no collapse or deformation and had a high printing accuracy of 97.37% as calculated using Equation 1. The printed structure showed good form retention with a printing accuracy of 93.64% even after standing for 30 min. As shown in Figure 3, it also demonstrated excellent adaptability in printing other complex, multi-dimensional models, such as small turtles and flower petals.

**Figure 3:**
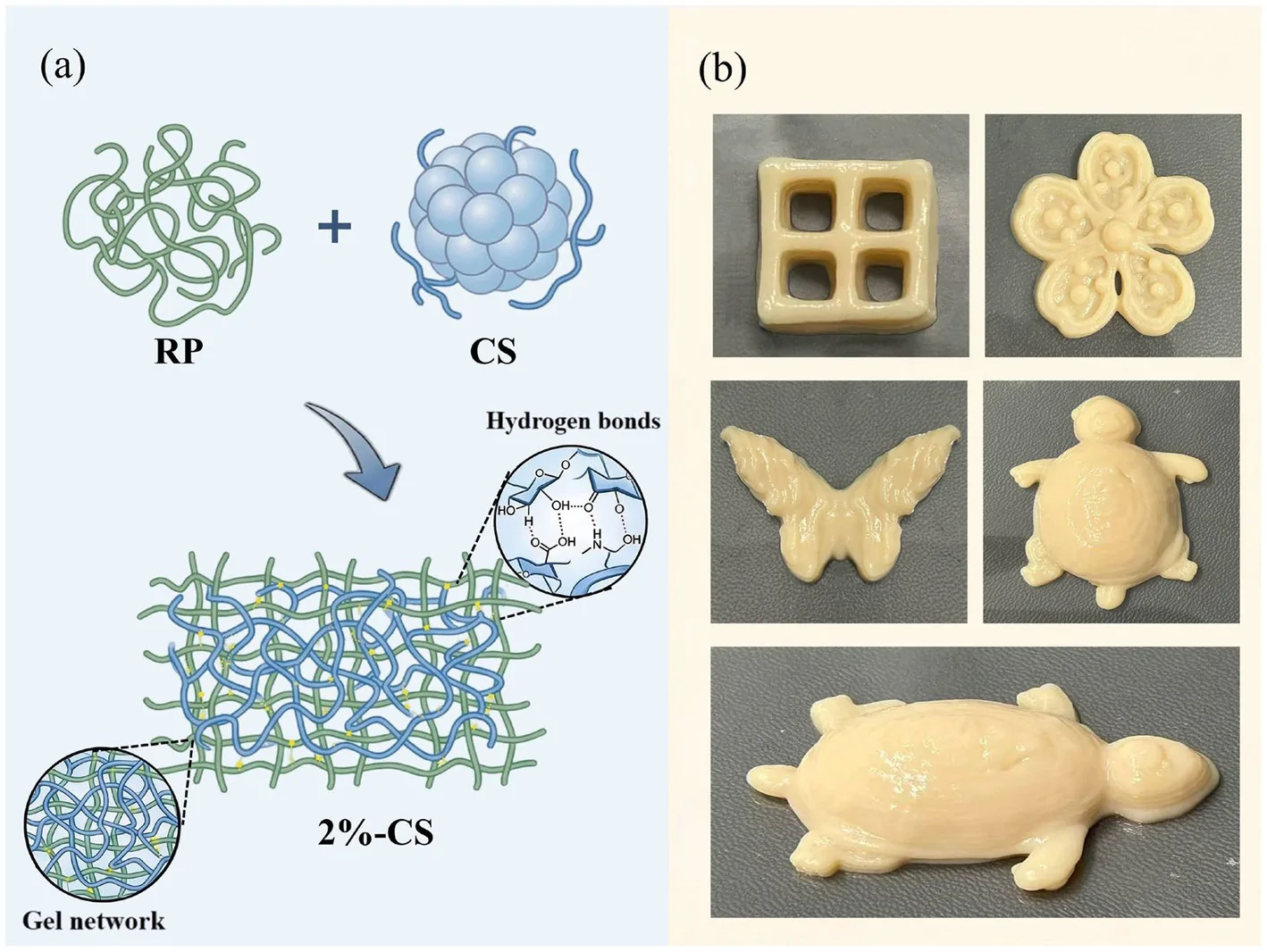
Characteristics of 2%-CS: **(a)** Molecular mechanism underlying the good printability of 2%-CS; **(b)** 3DP applications of 2%-CS after being left for 30 min in other models. Samples are labelled 0%-CS, 1%-CS, 2%-CS, 5%-CS, 7%-CS, 9%-CS, and 15%-CS, representing CS content of 0, 1, 2, 5, 7, 9, and 15% w/w in the CS-RP hybrid gels (total solids content 15% w/w).

The gel’s print accuracy decreased as CS concentration increased. The 7%-CS and 9%-CS samples had smooth edges and rough surfaces despite having good elasticity and stickiness. Because of its high viscosity, 9%-CS even clogged the printing nozzle. This led to disordered interlayer stacking, an uneven force distribution across the sample, and, eventually, a centre height of 9.14 mm, which was higher than the 8 mm model height. This suggests that excessive CS prevents a homogeneous gel network from forming, leading to an uneven composite gel surface, unusually high viscosity, and, eventually, decreased printing accuracy. This tendency was particularly noticeable in the pure CS gel 15%-CS, which had the lowest printing accuracy of 79.11%, despite its maximum viscosity. Similar phenomena have also been reported in the relevant literature by Li and Lou (25, 26), who found that excessively high polysaccharide content may negatively affect the printability of plant-based protein gels. Therefore, controlling the addition of polysaccharides is crucial for optimising the print quality of RP materials.

Because CS has a high amylopectin percentage (76%–85%) (27), no line breakage occurred in any sample. High amylopectin gels have been shown in earlier research to exhibit reduced yield stress, which promotes smooth ink extrusion (28). Additionally, amylopectin’s strong hydrophilicity prevented localised dehydration from causing line breaks during printing, allowing for continuous ink flow. Taken together, these factors result in the CS-RP composite exhibiting excellent extrusion stability and strong promise as a printing material.

### 3.2 Rheological properties

This study examined three rheological characteristics, including viscosity, modulus, and creep recovery, to determine the reasons for the collapse of 0%-CS during printing. Viscosity controls the ink’s smooth extrusion, modulus indicates its capacity for self-support, and creep recovery describes its resistance to deformation under sustained stress. The macroscopic variations seen in samples during 3DP can be thoroughly verified by measuring these three characteristics.

#### 3.2.1 Viscosity

The printing performance, molding precision, and extrusion stability of gel-based materials are all significantly influenced by viscosity. Excessively low viscosity results in inadequate structural stability, as the deposited ink lacks the mechanical strength to maintain its shape. Conversely, excessively high viscosity prevents ink from flowing, typically requiring higher extrusion pressure (29). To investigate further how shear rate affects gel viscosity, we used the power-law model with the expression η = K·γ^n-1^ to determine the shear rate exponent and the consistency index as given in Equation 2. The apparent viscosity of the ink is positively correlated with the K value; that is, a higher viscosity is often associated with a higher K value. A lower n number indicates a more pronounced shearthinning tendency in the system. The model’s goodness of fit is indicated by the R^2^ coefficient.

All of the created printing inks have n values less than 1, and as the shear rate increases, their viscosity decreases, as shown in Table 1 and Figure 4. This confirms that all gels exhibit the shearthinning behaviour required for 3DP. This consistency in n-values is significant, as lower n-values correspond to more pronounced shear-thinning behaviour, which lowers the bolus’s viscosity during oral processing and enhances both swallowing and palatability (30). According to Yang et al.’s study, the n-values of pea protein–carrageenan–starch composite gels ranged from 0.21 to 0.45, making them appropriate for people with difficulty swallowing (31). All composite gels in the current study exhibited n-values (0.16–0.43) consistent with those reported in the literature, indicating that they meet the sensory requirements for foods intended for people with dysphagia. 0%-CS exhibited significantly lower viscosity than all gels containing CS, as evidenced by the most severe collapse in its 3D-printed product. Increasing CS concentration markedly elevated ink viscosity, likely due to enhanced intermolecular connections and topological entanglement within the gel matrix, directly influencing rheological behaviour. Higher viscosity increases friction with the syringe, adversely affecting print outcomes. The 15%-CS sample had the highest K value (653.15 Pa·s^n^), indicating the highest viscosity, as indicated by the printing results. As a result, there was increased friction with the syringe, which eventually led to nozzle clogging and resulted in an uneven, rough surface on the printed sample. Conversely, gels with lower viscosity exhibit correspondingly smaller K-values, facilitating smoother extrusion from the syringe and achieving higher printing precision. This is also why 2%-CS exhibits the best printing performance among all gels, which is directly attributable to its lower, more suitable viscosity. This pattern is supported by the findings of Xu et al. (20), who reported that printing precision peaked at a K-value of 176.49 Pa·s^n^ for CS gel. In this study, the K value of the CS-RP hybrid gel at optimal printing precision (120.11 Pa·s^n^) is similar. Still, because rapeseed protein is introduced in this study system, the difference in K values between the two may be partly attributed to the modification of the protein component of the gel network structure. Nevertheless, the comparison initially shows that a moderate reduction in the K value improves the extrusion fluency of the composite gel, which is also a favorable condition for CS-2 to achieve high printing accuracy. Thus, adding RP significantly improves printing performance by altering the gel’s rheological properties.

**Table 1:** Power-law model parameters for different CS-RP hybrid gels.

Sample	K(Pa∙s^n^)	*n*	R^2^
0%-CS	21.96 ± 5.46^d^	0.31 ± 0.09^b,c^	0.999
1%-CS	115.86 ± 21.99^c^	0.22 ± 0.08^c,d^	0.997
2%-CS	120.11 ± 11.64^c^	0.18 ± 0.01^d^	0.996
5%-CS	272.53 ± 29.16^b^	0.16 ± 0.05^d^	0.989
7%-CS	273.57 ± 44.46^b^	0.28 ± 0.01^c^	0.996
9%-CS	275.48 ± 27.07^b^	0.40 ± 0.02^a,b^	0.992
15%-CS	653.15 ± 84.44^a^	0.43 ± 0.01^a^	0.963

Samples are labelled 0%-CS, 1%-CS, 2%-CS, 5%-CS, 7%-CS, 9%-CS, and 15%-CS, representing CS content of 0,1,2, 5, 7, 9, and 15% w/w in the CS-RP hybrid gels (total solids content 15% w/w).

**Figure 4:**
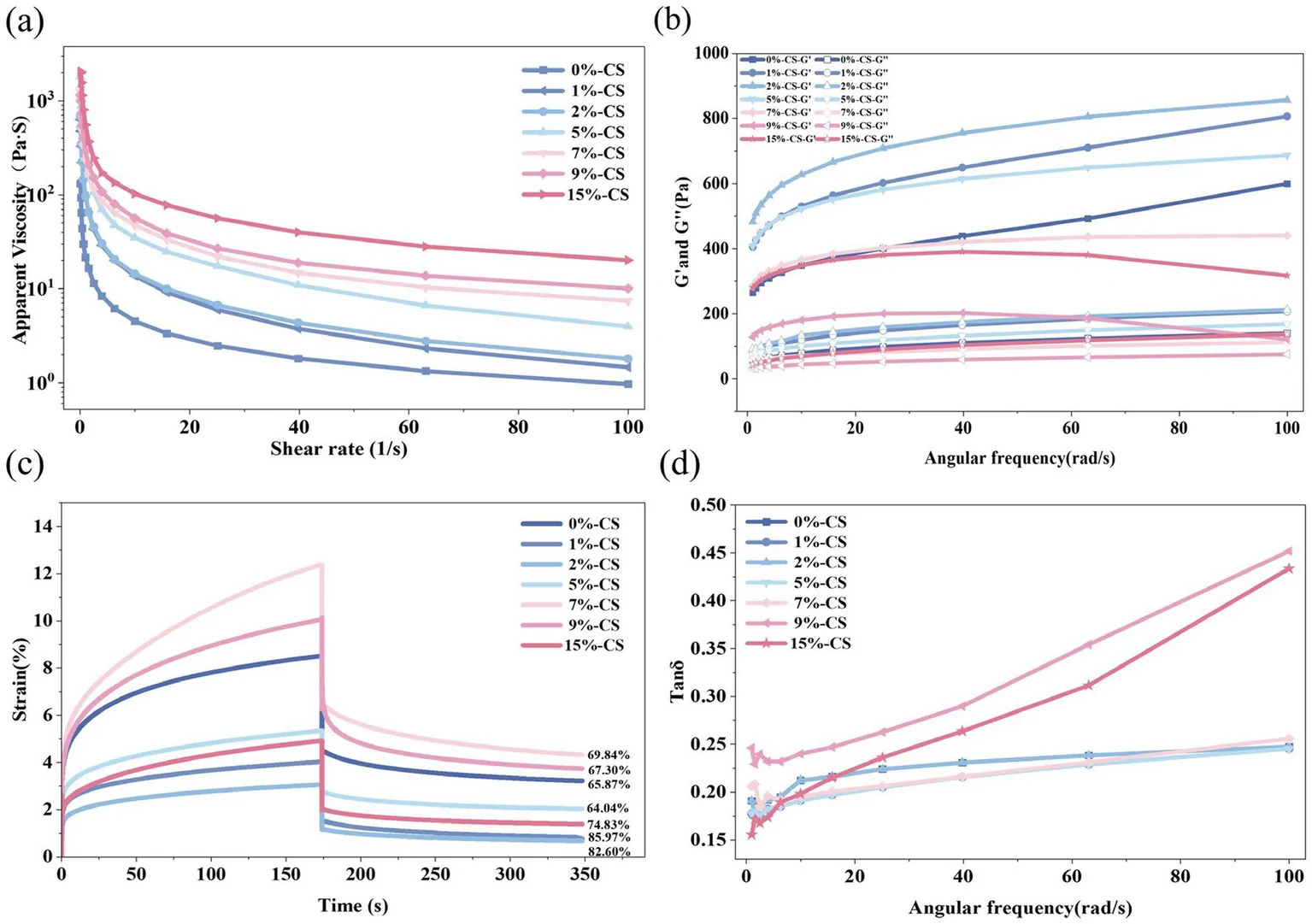
Rheological properties of CS-RP hybrid gels **(a)** Shear rate versus viscosity; **(b)** *G′* and *G″* versus angular frequency; **(c)** Creep recovery test; **(d)** *tan δ* versus angular frequency. Samples are labelled 0%-CS, 1%-CS, 2%-CS, 5%-CS, 7%-CS, 9%-CS, and 15%-CS, representing CS content of 0, 1, 2, 5, 7, 9, and 15% w/w in the CS-RP hybrid gels (total solids content 15% w/w).

#### 3.2.2 The dynamic rheological properties

The dynamic rheological properties revealed by frequency sweep experiments are closely related to the material’s self-supporting capability. It should be noted that the frequency sweep measurements characterize the frequency-dependent viscoelastic behaviour of the gel systems rather than their yield stress. The *G’* reflects the material’s elastic response, while the *G”* reflects its viscous response. Figure 4 shows that, for each gel sample, *G’* is larger than *G.”* Both *G’* and *G”* grow with frequency within the LVR, displaying typical solid-like properties. The material’s strong resistance to deformation is evident from the dominance of the elastic modulus, which is advantageous for the shape stability of items after 3DP (32). If the viscous modulus predominates, the material is more fluid and prone to collapse after printing.

Compared with 0%-CS, samples with 1% and 2% CS had higher *G’* and *G”* values. This conclusion is consistent with the findings of Xu et al. (33), which show that adding low-concentration polysaccharides can improve the gel’s mechanical strength and viscoelastic properties, which are essential for achieving high printing precision. As the CS concentration increases from 0% to 2%, the *G’ value* gradually rises, reaching a maximum. Typically, increased *G’* values result from stronger electrostatic interactions and hydrogen bonding between protein molecules and polysaccharide chains, leading to denser, more stable gel network structures (34). Greater resistance to deformation under external forces is indicated by higher *G’* values, which help guarantee that printed items retain the stability of their intended shapes. The optimal self-supporting capability of 2%-CS during printing, as evidenced by a high *G’* value of approximately 800 Pa, is a specific example of this. The WPI-XG system, which was likewise designed for easy-to-swallow foods, has a *G’ value* of 1,830 Pa (35), much higher than that of this system. This suggests that adding CS not only meets printing support requirements but also produces a softer gel texture that dissolves more easily in the mouth, making it a perfect material for creating foods that are easy to swallow. Further observation revealed that the *G’* declined, rather than continued to rise, as the CS concentration increased from 5 to 15%. This occurs because excess starch acts as a physical barrier, interfering with the orderly assembly of proteins at higher starch concentrations (36). Furthermore, pure CS has a lower modulus than pure RP gel, as 15%-CS has a lower modulus than 0%-CS. As a result, when CS takes over the system, the gel’s overall mechanical properties deteriorate, eventually lowering the gel modulus. Another critical rheological parameter that determines the self-supporting properties of printed structures is *tan δ*. Studies indicate that during swallowing, foods exhibiting solid-like characteristics, with *tan δ* values below 1, facilitate easier regulation of flow rate and smoother passage through the oesophagus. Results indicate that, except for the 9%-CS and 15%-CS samples, all samples meet the self-supporting region requirement (*tan δ* < 0.268) (37), with values below 1 and within the selfsupporting range. Consequently, all samples with CS concentrations below 9% demonstrate excellent edible delivery capability.

#### 3.2.3 Creep testing

Creep testing simulates the tongue’s propulsive action on food to evaluate a material’s shape retention under sustained loading and its rebound capacity after unloading (38). This test cannot be directly used to determine the yield stress or contact recovery characteristics of the material. During the creep stress phase, as depicted in Figure 4, all samples exhibit increasing strain over time, and the strain is irreversible. In general, the curves exhibit a comparable pattern. The final creep strain value of the gel, however, is where the differences reside. A material’s suppleness is often characterised by its creep-recovery compliance, which reflects its self-supporting behaviour in 3DP. In particular, lower recovery values indicate a more robust structure, whereas larger creep recovery values are associated with lower structural strength. Figure 4 shows the final strain values during creep in descending order, including 7%-CS, 9%-CS, 0%-CS, 5%-CS, 15%-CS, 1%-CS, and 2%-CS. The final strain value measured by 2%-CS during the creep stage was 3.06%, lower than that of the other samples as calculated using Equation 3. This result shows that 2%-CS has the strongest anti-deformation ability under a constant stress of 10 Pa, which may help it maintain good structural integrity during 3D printing deposition. The addition of CS has a concentration-dependent influence on the gel network. Moderate physical entanglement between polymer chains develops at CS concentrations of 1%–2%, thereby increasing network density and strengthening the gel structure. The final strain value increased as the CS concentration climbed to 7% and 9%. Fan’s research (39) and subsequent FT-IR studies indicate that there are no covalent bonds between CS and RP. The protein is therefore dispersed in a starch-predominant phase (39). Gel stability weakens as the effective crosslinking density per unit volume decreases, even though the protein’s intrinsic disulfide bonds remain intact.

After stress removal, the strain gradually decreases during the recovery phase but does not reach zero, indicating permanent deformation. This indicates that the gel behaves viscoelastically, consistent with the previously described analysis of the modulus data. By measuring the percent recovery after stress removal, we assess the gel’s capacity to repair its structure. Therefore, a lower final creep strain indicates greater deformation resistance, while a higher recovery rate indicates stronger structural recovery capacity. In contrast to 0%-CS (65.87%), 15%-CS (74.83%), and the other three hybrid gels, the percent recovery values for 1%-CS and 2%-CS samples were 85.97 and 82.60%, respectively, as indicated in Figure 4. The gel’s capacity to retain printed shapes and avoid collapse is directly enhanced by its strong structural recovery.

Notably, although providing improved structural stability throughout 3DP, materials with lower strain levels require greater force to rearrange the food bolus during swallowing (40), which presents challenges for those with dysphagia. This contradiction emphasises the need to balance robust structural support with the capacity to permit mild bolus deformation during swallowing when creating 3DP inks for dysphagia diets.

### 3.3 Texture properties

The rheological parameters determined above reflect the material’s instantaneous response behaviour under stress. To further investigate the texture properties of these samples, we carried out TPA testing. The TPA test simulates the oral chewing process by subjecting the test material to two successive compression cycles, thereby quantifying and evaluating its textural qualities. Key criteria examined in this study include cohesiveness, hardness, chewiness, and adhesiveness, as depicted in Figure 5. Cohesiveness and hardness indicate material stability, while chewiness and adhesiveness closely mimic the mechanical responses samples experience in the oral cavity.

**Figure 5:**
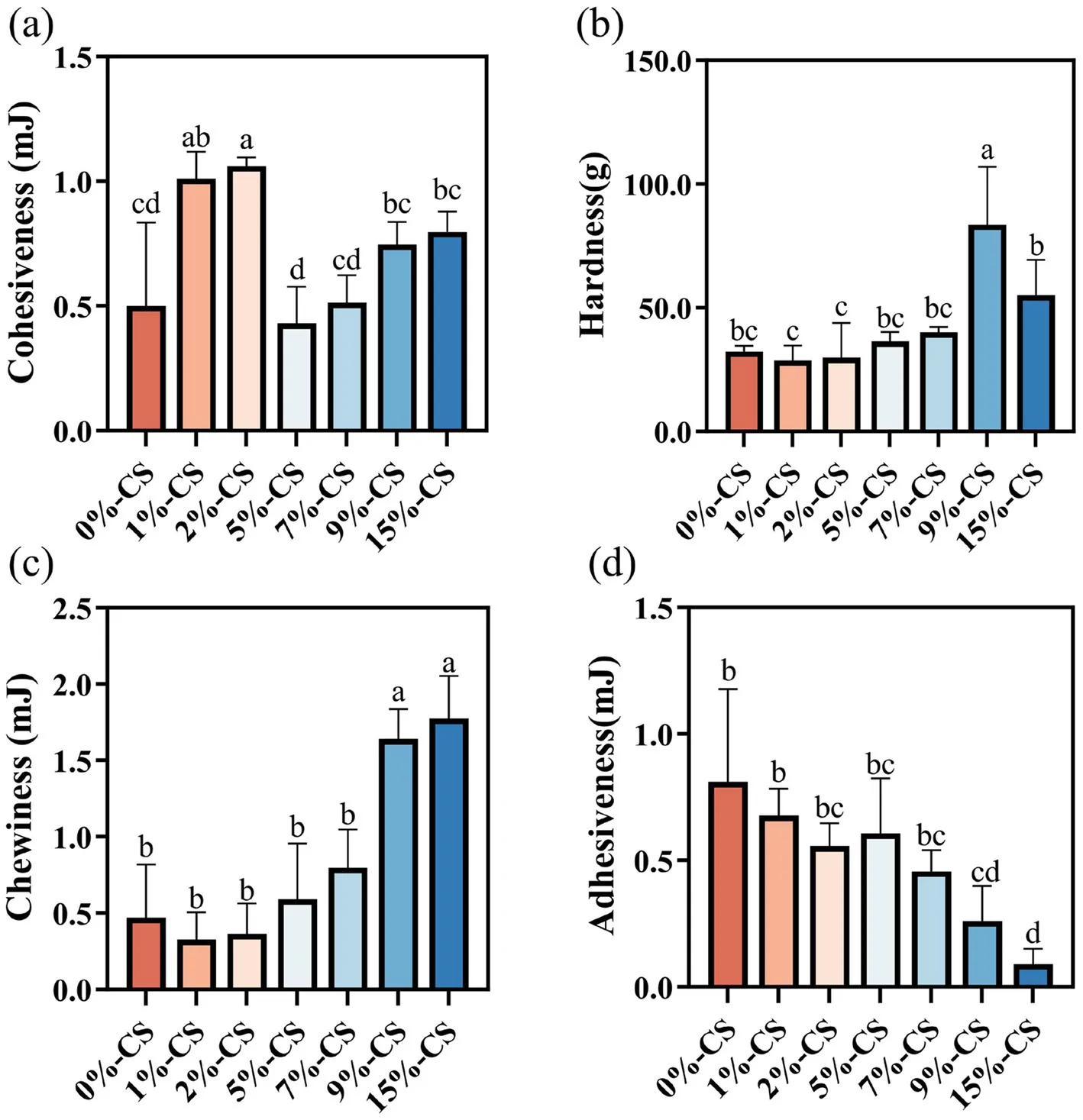
Texture properties of each formulation at different CS concentrations: **(a)** Cohesiveness; **(b)** hardness; **(c)** chewiness; **(d)** and adhesiveness. The error bars represent the standard deviation of the mean. Different letters indicate statistically significant differences between samples (*p* < 0.05). Samples are labelled 0%-CS, 1%-CS, 2%-CS, 5%-CS, 7%-CS, 9%-CS, and 15%-CS, representing CS content of 0, 1, 2, 5, 7, 9, and 15% w/w in the CS-RP hybrid gels (total solids content 15% w/w).

Cohesiveness is a criterion for evaluating the internal structural strength of food. Generally, meals with high cohesiveness tend to retain their shape during chewing and swallowing, which is particularly beneficial for people with dysphagia. At 1.06 mJ, the 2%-CS formulation showed the strongest cohesiveness among the CS-containing groups. This Figure 5 satisfies the performance requirements for foods that are easy to swallow, as it is equivalent to the 1.27 mJ cohesiveness reported by Xiao et al. (41) for a 3% soy protein-white mushroom powder gel. When the CS concentration exceeds 5%, cohesiveness first decreases and then gradually increases from 0.43 mJ to 0.80 mJ, indicating that the intramolecular interactions within the composite gel system are relatively complex. Another crucial measure of structural stability, alongside cohesiveness, is hardness. Hardness indicates the force needed to chew food as well as the degree of internal matrix breakdown; excessive hardness makes chewing more difficult for older people. The hardness of all samples was less than 85 g, which is below the 89.21 g safety criterion for mashed potatoes documented in the literature (42) for dysphagia diets. It is clear from comparing the hardness of the 0%-CS and 15%-CS samples that CS is primarily responsible for the increase in hardness, while the total solids content remains 15%. The main reason for this increase in hardness is that the addition of CS alters the gel microstructure, making the network stronger and more rigid. Chewiness has been consistently reported to be positively correlated with hardness in numerous studies (43). This association was confirmed across all study samples. Chewiness increased progressively with increasing carbohydrate concentration and hardness. Specifically, the chewiness of 2%-CS was measured at 0.36 mJ, which is lower than that of soy yoghurt (1.80 mJ) reported in the literature (44). This suggests that the mouth needs less energy to break it down into an easily ingested bolus. Adhesiveness assesses swallowing safety by predicting whether food may adhere to the oesophagus during swallowing. For older people with dysphagia, semi-solid meals with high adhesiveness considerably increase their swallowing difficulty. Lee and colleagues categorised food adhesiveness into three distinct levels: Level 1 (<4 mJ), Level 2 (4–18 mJ), and Level 3 (>18 mJ). Increased food adhesiveness is associated with a higher likelihood of pharyngeal residue post-swallowing (45). Specifically, Level 1 adhesiveness is associated with the lowest residue risk, whereas higher levels are associated with a higher residue risk. The samples are suitable for patients with dysphagia because, as Figure 5 illustrates, their adhesiveness varies minimally across varied concentrations and falls within Level 1. Notably, the cohesiveness of the 2%-CS sample was much higher than that of the other sample sets, and its hardness was significantly lower than that of the 15%-CS sample. In addition to its soft texture, which is easy for older people to chew, this feature indicates that the structure can effectively withstand external stress, allowing for the gradual release and absorption of nutrients (46). It is also regarded as the most promising easy-to-swallow food (47). These findings suggest that the gel’s textural characteristics can be precisely adjusted to meet individual requirements by controlling the CS: RP ratio.

### 3.4 Moisture distribution

LF-NMR was used to analyse peak regions and relaxation times, allowing us to study the microscopic water distribution in CS-RP hybrid gels. The relaxation time spectra often show three distinct peaks: bound water (T₂₁), semi-bound water (T₂₂), and free water (T₂₃). All CS-RP hybrid gels showed notable peaks at T_22_, with T_22_ peak area contributions above 90%, as Figure 6 and Table 2 illustrate. This verifies the prominent role of semi-bound water in the gel’s hydration system, indicating a stable network structure and favourable gel characteristics.

**Figure 6:**
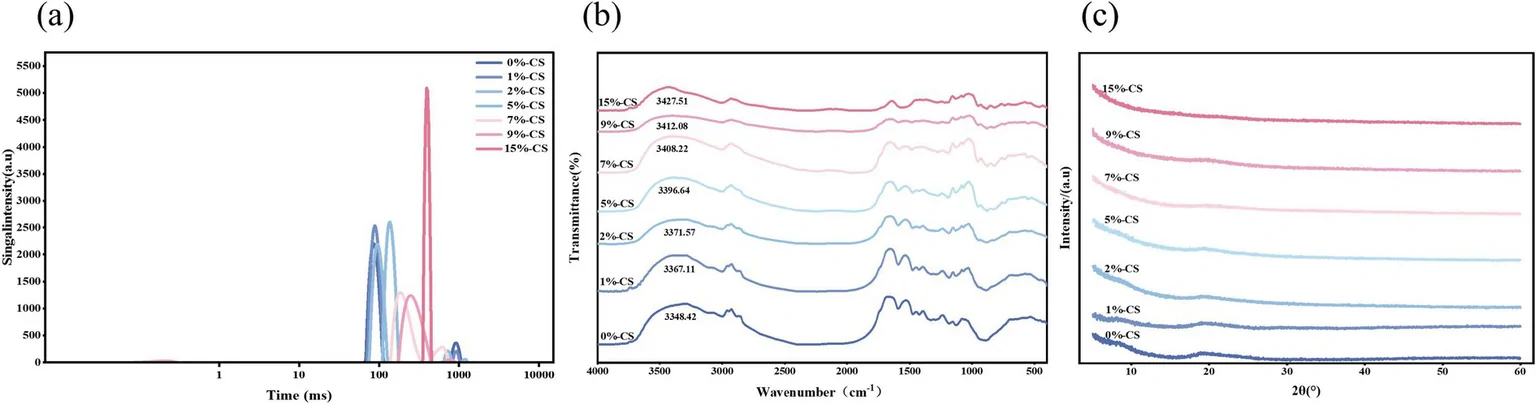
Microscopic characterization of CS-RP hybrid gel: **(a)** Moisture distribution images; **(b)** Infrared spectrum at 400–4,000 cm^−1^; **(c)** Crystal structure. Samples are labeled 0%-CS, 1%-CS, 2%-CS, 5%-CS, 7%-CS, 9%-CS, and 15%-CS, representing CS content of 0, 1, 2, 5, 7, 9, and 15% w/w in the CS-RP hybrid gels (total solids content 15% w/w).

**Table 2:** Relaxation time versus peak area percentage for all CS-RP hybrid gels.

Sample	T_22_(ms)	T_23_(ms)	P_22_(%)	P_23_(%)
0%-CS	81.12 ± 7.55^d^	701.58 ± 343.98^a^	91.40 ± 2.09^b^	8.603 ± 2.09^b^
1%-CS	84.01 ± 4.78^d^	830.35 ± 81.19^a^	93.18 ± 3.79^a,b^	6.82 ± 3.79^b,c^
2%-CS	92.08 ± 3.87^d^	839.98 ± 106.31^a^	96.92 ± 1.86^a,b^	3.08 ± 1.86^b,c,d^
5%-CS	133.16 ± 3.69^c^	862.24 ± 754.37^a^	99.51 ± 0.48^a^	0.49 ± 0.48^d^
7%-CS	196.35 ± 10.87^b^	450.40 ± 395.48^a^	92.50 ± 7.43^a,b^	6.99 ± 6.68^b,c^
9%-CS	137.47 ± 35.49^a^	1225.38 ± 727.30^a^	96.83 ± 2.38^a,b^	1.35 ± 0.75^c,d^
15%-CS	/	407.74 ± 37.07^a^	/	100.00 ± 0.00^a^

Different letters indicate statistically significant differences between samples (*p* < 0.05). Samples are labelled 0%-CS, 1%-CS, 2%-CS, 5%-CS, 7%-CS, 9%-CS, and 15%-CS, representing CS content of 0, 1, 2, 5, 7, 9, and 15% w/w in the CS-RP hybrid gels (total solids content 15% w/w).

A shortened relaxation time typically indicates tighter bonding between water molecules and the gel network, resulting in reduced water mobility (48). As shown in Table 2, the control group without CS (0%-CS) had the shortest relaxation time (81.12 ms) among all samples, suggesting that pure RP gel has a relatively strong ability to restrain water. The relaxation time of 1%-CS and 2%-CS rose to 84.01 ms and 92.08 ms, respectively, upon the incorporation of trace quantities of CS. The original RP interactions were altered by the addition of CS, which increased the mobility of some water molecules. Nevertheless, the gel’s structural integrity was unaffected by this slight increase in water mobility. Intermolecular interactions between RP and CS molecules resulted in a denser, more homogeneous composite network, as evidenced by TPA and FT-IR measurements. This greatly improved the gel’s cohesiveness while preserving its low hardness. Therefore, this structural feature of 1%-CS and 2%-CS effectively lowers the risk of material dispersion-induced choking during swallowing. The T_22_ peak gradually shifts to the right as the CS concentration increases. Although gelatinized CS has strong water absorption and retention properties, excessive CS may alter the spatial structure of the RP-CS composite network, weaken the effective connections between RP molecules, and make the network more loosely packed, thereby reducing the system’s overall binding ability to water. Therefore, the increase in water mobility in the system is more likely due to the reconstruction of the composite network caused by excessive CS, rather than to the lack of water retention capacity in CS itself. When CS occupies the entire solid phase of the system, all bound water is converted to free water. This suggests that the mobility of the dominant semi-bound water is continuously increasing while the gel network’s capacity to hold water is deteriorating. This mechanism contrasts with the mechanism reported in the literature for the release of free water from starch upon the addition of casein (49). According to Ji’s research, highly hydrophobic casein released free water by interfering with starch rearrangement and crystallisation through adsorption and competitive effects (49). In the current system, adding surplus CS, which has a limited water-retention capacity, may compromise the integrity of the RP network by competing for water. This reduces the gel’s capacity to retain moisture, thereby increasing its fluidity.

Although, in general, shorter relaxation times are associated with more accurate 3DP performance (50), the above results show no simple linear correspondence between the fluidity of water molecules, as reflected by LF-NMR in this system, and 3D printing performance. Although the 0%-CS sample shows the shortest T2 (81.12 ms) among all samples and exhibits strong water retention capacity, the effective connectivity and bearing capacity of its single network may be insufficient, making it difficult to maintain the sedimentary structure after printing. In contrast, although the T2 of 1%-CS and 2%-CS has increased slightly, the introduction of CS may improve the structure of the RP network and its macromechanical properties, thereby enhancing the gel’s self-supporting ability. Thus, at the microscopic moisture level, LF-NMR offers evidence linking textural characteristics and 3DP performance; however, interpretation requires integration with macroscopic mechanical indicators, such as rheological parameters.

### 3.5 Intermolecular interactions

To better understand the gel network structure and investigate intermolecular forces in the CS-RP system, FT-IR spectroscopy was used. In line with the findings of Min et al. (51), the CS-RP sample showed no additional absorption peaks compared with 0%-CS and 15%-CS, as shown in Figure 6. This suggests that no new covalent bonds or chemical interactions were produced by the addition of RP. The stretching vibration of hydrogen bonds formed between hydroxyl groups was observed as a broad, significant band in the range 3,000–3,600 cm^−1^ across all samples. With the increase in CS concentration, the O–H telescopic vibration peak at about 3,348 cm^−1^ tends to shift to a higher wavenumber, suggesting that hydrogen bonding in the system may be weakened and accompanied by reorganisation of the hydrogen-bond network; this effect is in line with Liu et al.’s (52) findings that high concentrations of xanthan gum reduce intramolecular hydrogen bonding in pea protein. Notably, Jiang et al.’s (53) research demonstrated that hydrogen bonds remain crucial in protein-polysaccharide noncovalent complexes despite the rearrangement of the hydrogen-bond network. Therefore, the network topology of the composite gel created by CS and RP depends on hydrogen-bond interactions.

### 3.6 Crystal structure

The crystalline and amorphous structures of the materials were characterised by XRD. The XRD pattern of the CS-RP composite gel is shown in Figure 6. The XRD pattern of the CS-RP hybrid gel in this study showed no clear, sharp diffraction peaks, except for narrow peaks near 6° and 18°. It shows that the ordered component accounts for only a very small proportion of the overall gel structure. Therefore, the CS-RP composite gel can be described as a system with an ordered structure, mainly in an amorphous state, and containing trace residues. Ji et al.’s findings on rice proteinstarch composite gels, which revealed that rice starch transformed from its original Type A structure to a fully amorphous state after heat gelatinisation (54), are consistent with this observation. Furthermore, the XRD results are supported by the FT-IR data, which indicate that the interactions between CS and RP are primarily noncovalent. The predominant hydrogen-bonding interactions also disrupt the original crystalline arrangement, forming an amorphous gel.

### 3.7 Microstructural characteristics

We used SEM to characterise the morphology of various samples. Microstructural alterations in CSRP hybrid gels at different CS concentrations are seen in Figure 7. 0%-CS had the coarsest and most irregular pore-size distribution, which likely led to poor 3D printability and reduced macroscopic mechanical properties. On the other hand, due to the gelatinisation of CS, 15%-CS exhibited a dense honeycomb network structure (55).

**Figure 7:**
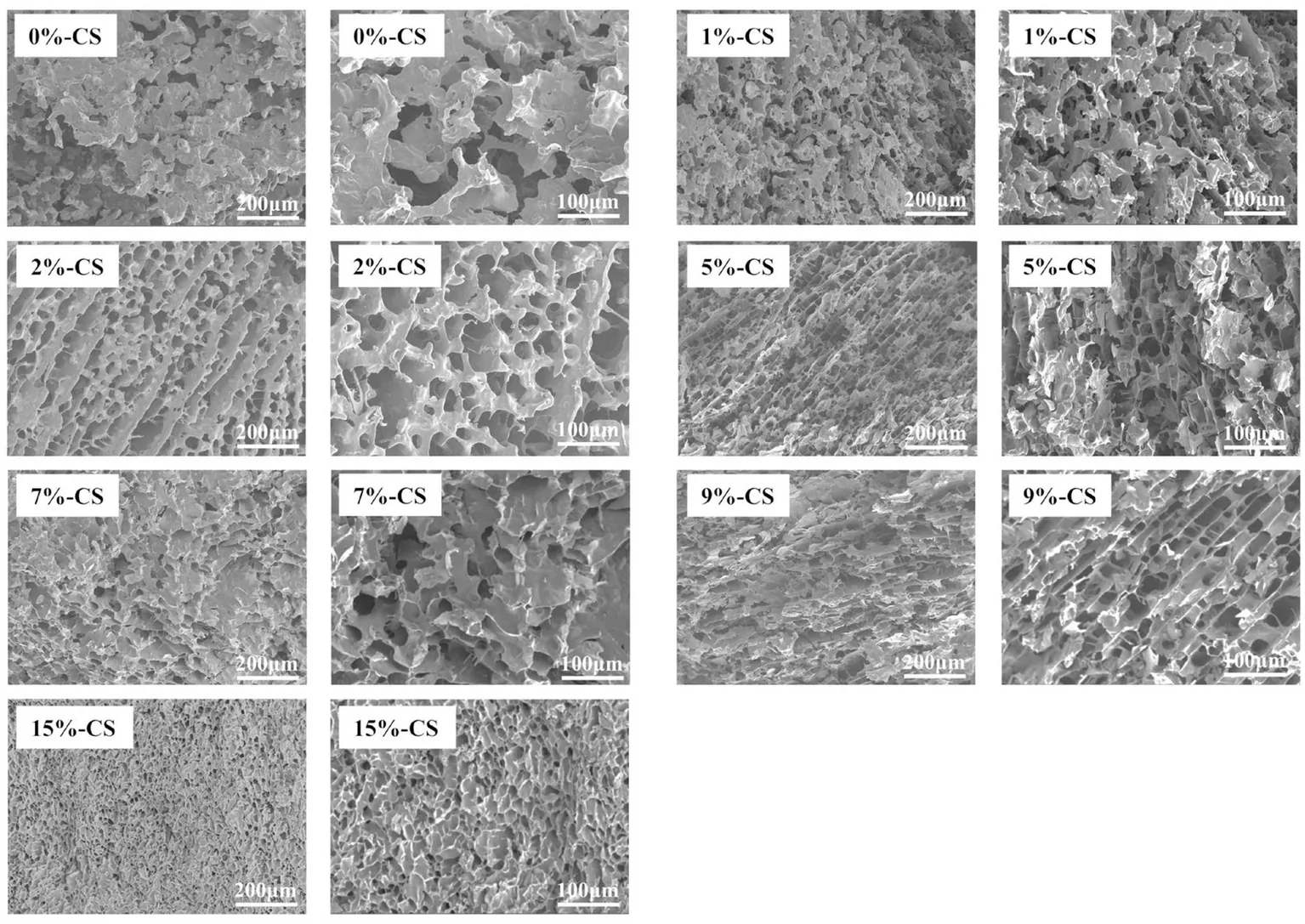
SEM images of different CS-RP hybrid gels (scale bar = 100 μm, 200 μm). Samples are labelled 0%-CS, 1%-CS, 2%-CS, 5%-CS, 7%-CS, 9%-CS, and 15%-CS, representing CS content of 0, 1, 2, 5, 7, 9, and 15% w/w in the CS-RP hybrid gels (total solids content 15% w/w).

According to SEM data, the gel network’s shape, pore-size distribution, and structural density are all significantly affected by the addition of CS. Every sample has a reticular (porous) layered microstructure, as shown in Figure 7. The network structure became denser, and the pore size steadily shrank as the CS concentration rose from 1% to 5%; at 5% CS, the network structure became more noticeable. This was mostly because more CS encouraged RP to create cross-links. To maintain support strength during layer-by-layer deposition and improve printing accuracy, studies have demonstrated that a sufficiently dense network structure enhances the material’s mechanical response and encourages the formation of uniform, continuous extruded filaments (56). Nevertheless, a clear layered structure progressively appeared in the gel when the CS content was raised to 7% and 9%. Weakened intermolecular interactions result from the porous structure’s stability being affected by the transition from a porous network to a layered structure (19). This structural evolution reduces the gel network’s structural support capability, resulting in a considerable drop in printing accuracy for 7%-CS and 9%-CS.

The above SEM observation is primarily a qualitative description, focusing on the overall evolution of the microscopic morphology across different formulas. Quantitative aperture analysis was not performed due to the uneven distribution of pore sizes in the sample and the lack of standardized methodological standards. Nevertheless, the consistent trend observed across the concentration gradient provides clear evidence of CS addition’s regulatory effect on the gel network’s structure.

### 3.8 Assessment of swallowability

Foods linked to dysphagia are identified and categorised using the IDDSI. We tested each gel sample using IDDSI to characterise it from a swallowing safety perspective, as shown in Figure 8. No whitening appeared on the nail for any sample, as seen in Figure 8, which is consistent with the findings reported by Xing et al. (57) for their black fungus-xanthan gum gel samples. The fork mark on 15%-CS was nearly undetectable, while the indentations on 0%-CS and 1%-CS samples were blurry after the fork was removed. At a concentration of 5% CS, a minor quantity of material was noted to be displaced at the juncture of contact between the forks. There were noticeable fork marks in the 7%-CS sample because a lot of material was dragged with the fork.

**Figure 8:**
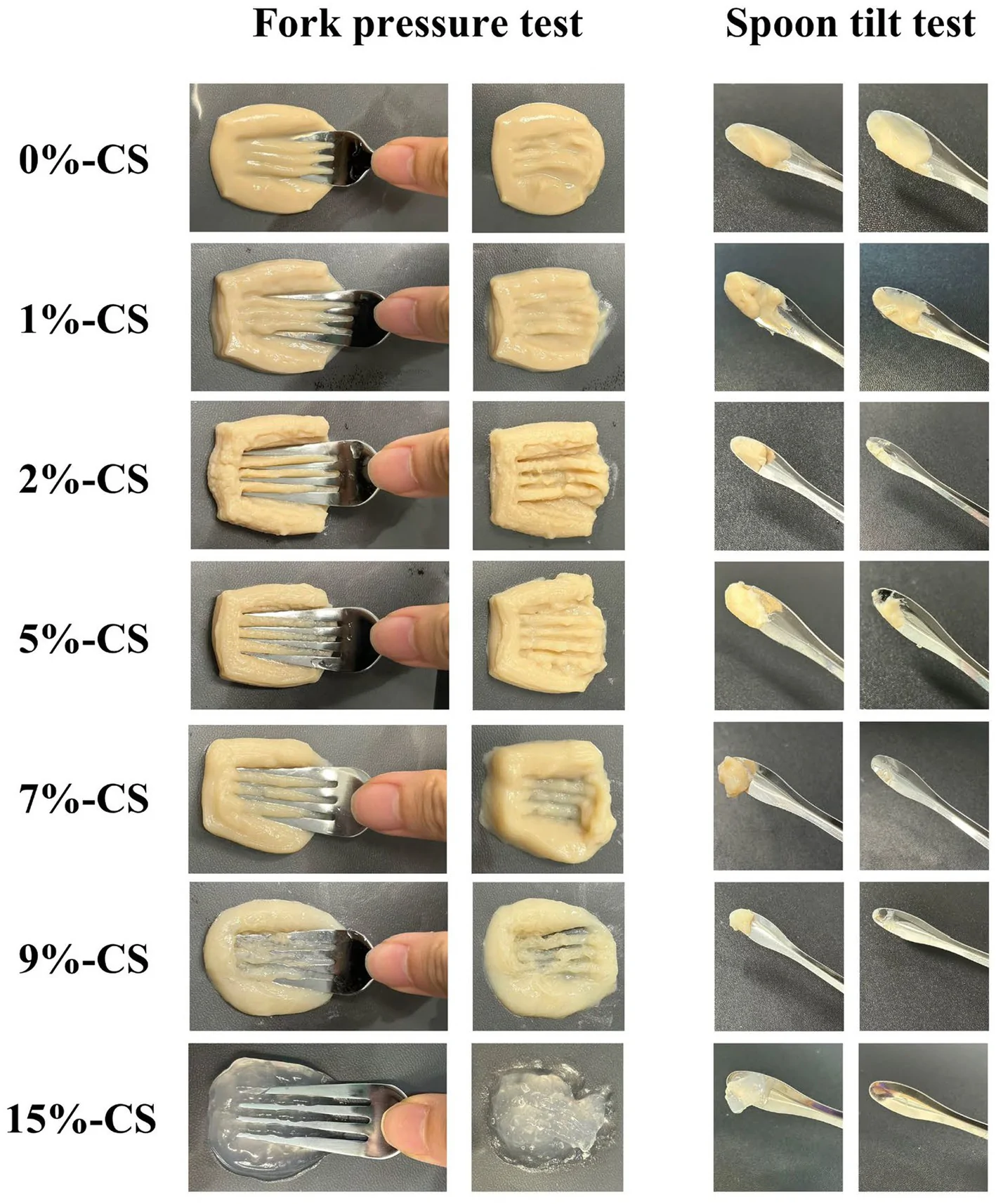
IDDSI testing of all CS-RP hybrid gels. Samples are labelled 0%-CS, 1%-CS, 2%-CS, 5%-CS, 7%-CS, 9%-CS, and 15%-CS, representing CS content of 0, 1, 2, 5, 7, 9, and 15% w/w in the CS-RP hybrid gels (total solids content 15% w/w).

The spoon tilt test shows the adherence and cohesivity of a sample. This cohesivity differs from the cohesiveness trait observed in the samples’ textural properties (41). Results indicated that at CS concentrations of 0%, the samples adhering to the spoon were strong, with negligible detachment when the spoon was tilted. Although 0%-CS exhibited the highest cohesiveness in the TPA test, its adhesiveness reached a maximum of 0.81 mJ, indicating resistance to slippage. Similarly, samples with a 1% CS concentration demonstrated strong adhesion in the tilt test. However, from a swallowing safety perspective, its tendency to stick to the spoon surface may keep the sample in the mouth, potentially causing aspiration. Therefore, it is unsuitable for patients with dysphagia. At CS concentrations of 2, 5, 7, 9, and 15%, minimal residue remained, allowing samples to detach cleanly from the spoon. The gel with a 15% CS concentration was the most fluid, as indicated by the lowest adhesion value of 0.09 mJ among all samples and the least residue on the spoon. This indicates that CS has a lower water-holding capacity than RP, consistent with LF-NMR results. It can thus be concluded that composite gels with a CS concentration ranging from 2% to 9% not only possess adequate cohesive strength to maintain their structure when used as food but also effectively prevent residue from remaining in the mouth, thereby meeting the texture requirements for Level 4 foods under the IDDSI framework (58). Therefore, we may adapt diets for patients with varied degrees of dysphagia by adjusting the concentration of CS.

### 3.9 Effects of CS content on microstructure and textural properties of RP

Figure 9 shows the regulation mechanism of CS in RP gel systems in conjunction with the experimental findings mentioned above. Building on the analysis of CS content’s effects on RP microstructure and texture, we next examined the effects of various CS: RP ratios on the macroscopic characteristics of the samples, while maintaining a total solid content of 15% w/w. In particular, the RP-based gel’s ease of swallowing and 3DP ability are both successfully modulated by the addition of CS. *G′* and the percent recovery are significantly improved when CS is present at an ideal concentration because it decreases water mobility and encourages non-covalent cross-linking among amorphous molecules, mostly through hydrogen bonding. Extrusion from the printing nozzle is facilitated by increased amylopectin concentration and reduced viscosity. On the other hand, excessive CS addition leads to over-crosslinking between CS and RP, creating an overly dense, viscous network that, in turn, causes several 3DP performance problems. Composite gels containing 2–9% CS successfully altered the RP-based gel’s textural characteristics in swallowability tests, imparting superior cohesiveness, adhesiveness, and hardness. This substantially reduced the likelihood of the gel adhering to the neck or oesophagus in elderly people. These results show that hybrid gels have great promise for easily swallowed foods. It is important to note that the 2%-CS gel can achieve high gel strength and swallowing safety without sacrificing printing accuracy, making it ideal for quickly preparing customised meals for people with dysphagia in senior care environments.

**Figure 9:**
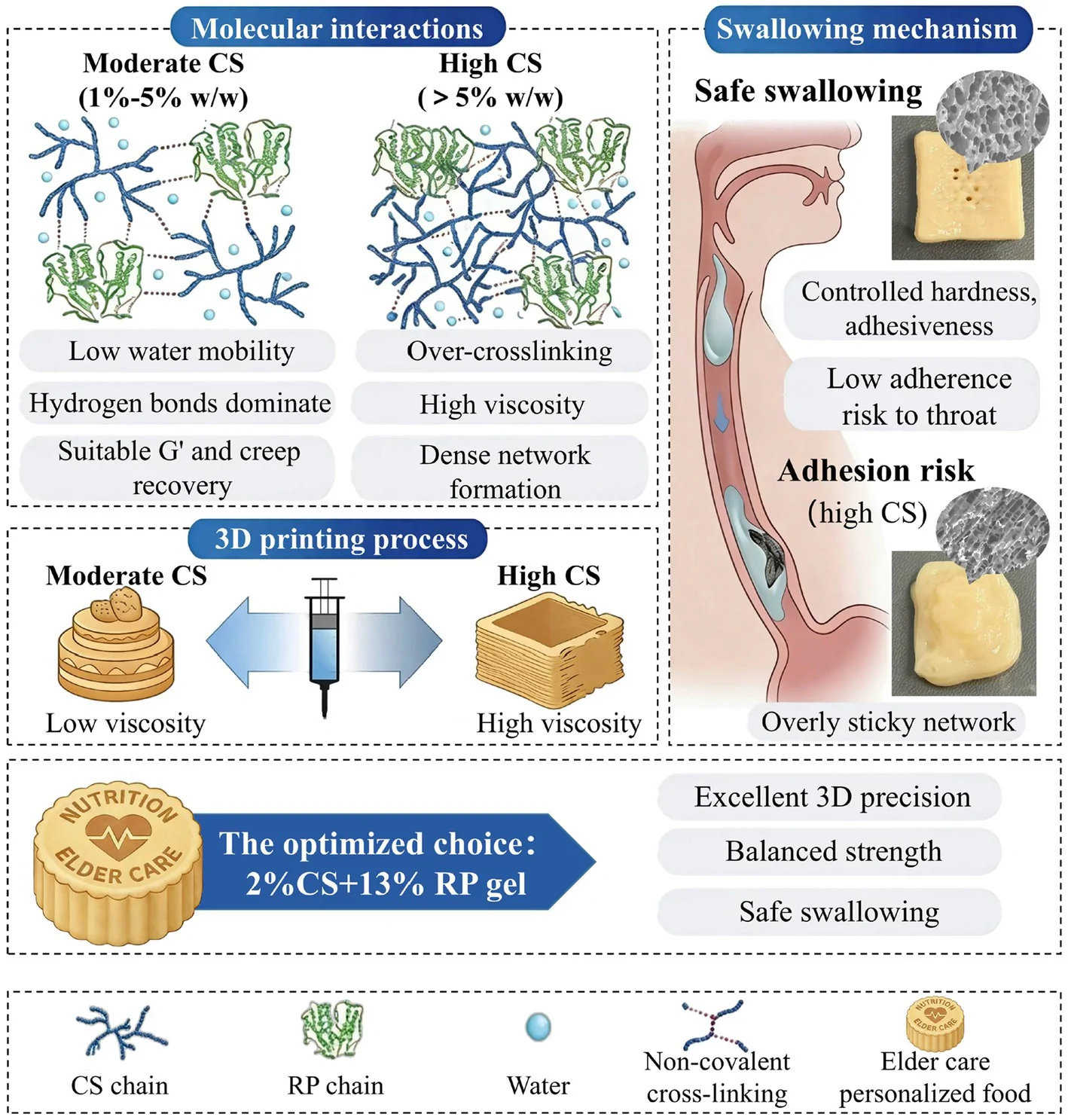
The way that CS affects the microstructure and texture characteristics of RP.

## 4 Conclusion

In this work, we gradually varied the CS: RP ratio while maintaining a constant total solids content of 15% w/w to comprehensively examine the effects of CS content on the printability and swallowability of RP. According to rheological and textural data, 1–5% CS can give the gel the right amount of cohesiveness and hardness while maintaining its ability to support itself. The gel demonstrated the best combination of 3D printability and ease of swallowing at 2% CS, and was categorised as IDDSI Level 4 due to its lower apparent viscosity, higher *G′*, and higher creep recovery rate. According to preliminary SEM, FT-IR, XRD, and LF-NMR investigations, the contact mechanism between CS and RP is non-covalent and mainly involves hydrogen bonding, with 1–5% CS exhibiting good water-retention capability. In conclusion, CS concentrations below 5% can serve as an efficient polysaccharide component to enhance the textural, swallowing, and printing qualities of RP in a synergistic manner. This study promotes the industrialisation of 3D printing technology for easy-to-swallow speciality meals and provides a theoretical foundation for the use of CS-RP hybrid systems in 3D food printing inks. The sensory acceptability of CS-RP hybrid gels in patients with swallowing difficulties will be further examined and improved in future studies.

## Data Availability

The original contributions presented in the study are included in the article/supplementary material, further inquiries can be directed to the corresponding author.
